# Neurotoxic Effects of Nanoplastics: Current Evidence and Mechanistic Insights

**DOI:** 10.3390/toxics14050387

**Published:** 2026-04-30

**Authors:** Fuxin Wan, Xiaohan Liu, Jiayue Pan, Linping Ke, Zhekai Zheng, Jingling Liao

**Affiliations:** Academy of Nutrition and Health, Hubei Province Key Laboratory of Occupational Hazard Identification and Control, School of Public Health, Wuhan University of Science and Technology, Wuhan 430065, China

**Keywords:** nanoplastics, neurotoxicity, toxic mechanism, neurodegenerative diseases, neuroinflammation, oxidative stress

## Abstract

Plastic products are extensively utilized in various industrial goods and consumer items. However, when these plastics fail to undergo complete degradation, they generate nanoplastic particles (NPs). As emerging environmental pollutants, such nanoplastics are highly likely to have widespread and adverse impacts on human health. Accumulating evidence indicates that NPs can penetrate biological barriers and exert toxic effects on multiple organs, including the nervous system. Although extensive studies have investigated the toxicity of NPs, the mechanisms underlying their long-term neurotoxic effects remain poorly understood. Here, we summarize the current understanding on the exposure pathways of NPs, their neurotoxic effects, and the molecular mechanisms involved in neurotoxicity. Emerging evidence suggests that NPs induce neurological damage through various mechanisms, including oxidative stress, neuroinflammation, ferroptosis, autophagy dysregulation, and gut–brain axis imbalance. A comprehensive understanding of these mechanisms will provide new insights into the potential impacts of environmental NPs exposure on the nervous system and contribute to more accurate health risk assessments.

## 1. Introduction

In recent years, the production of plastic products has shown a steady increase annually. Among materials, polystyrene is one of the most widely used plastics, with extensive applications in both industrial and daily consumption [1]. Through various environmental processes—including mechanical degradation, ultraviolet radiation, and biological decomposition—these plastic products are ultimately progressively fragmented into microplastics (MPs) and nanoplastics (NPs) [2,3]. In fact, NPs can be subdivided into numerous categories based on their plastic sources, including polyethylene (PE), polypropylene (PP), polyvinyl chloride (PVC), polystyrene (PS), polyethylene terephthalate (PET), and polyamide (PA), among others. Due to differences in molecular structure and chemical composition among various types of nanoplastics, they often exhibit significant variations in environmental transport and transformation characteristics, bioavailability, and potential toxic effects. For instance, polyethylene nanoplastics typically exhibit excellent chemical stability and are resistant to degradation in natural environments, making them prone to persist in water bodies and soils for extended periods. In contrast, polyvinyl chloride nanoplastics, due to the chlorine atoms in their molecular chains, may release hazardous substances under specific conditions, posing additional potential threats to ecosystems and organism health. These nanoparticles, derived from different types of plastics, after entering the environment through various pathways, interact with surrounding substances, thereby exerting complex and diverse impacts on both the environment and living organisms. Plastic particles can exert diverse and multifaceted adverse effects on the ecological environment and living organisms. It is noteworthy that although people are exposed to a wide variety of NPs in daily life, laboratory studies on nanoplastics toxicity still primarily focus on polystyrene nanospheres with different characteristics than the research subject.

Currently, organisms are exposed to NPs mainly through three pathways. Oral ingestion is the most significant pathway, occurring when humans consume contaminated food or water [4]. The average polystyrene particles content detected in plastic drinking bottles exceeds one hundred thousand, with approximately 90% of these particles at the nanoscale. Inhalation via the respiratory tract is particularly prominent in industrialized cities, where airborne NPs can enter the body through the nasal cavity and lungs. Although dermal penetration of NPs is relatively limited, NPs may still enter the organism through damaged skin or hair follicles following contact with personal skincare products or industrial materials containing NPs [5].

NPs with diameters of less than 1 μm possess a large specific surface area and enhanced biological permeability, enabling them to more readily penetrate biological barriers, enter cells, disrupt organelles, and interfere with cellular homeostasis [6]. These properties can lead to tissue damage and a range of toxicological effects, making NPs a major focus of current research. However, it is of great concern that existing studies have successfully detected the presence of plastic particles in human brain tissue [7]. Furthermore, relevant studies have revealed that the concentration of nanoplastic particles in the brain tissues of dementia patients (including types such as Alzheimer’s disease and vascular dementia) is significantly higher than that in the control group with normal neurological function [8]. This groundbreaking discovery holds significant implications, as it directly demonstrates that NPs not only possess the ability to breach the crucial physiological barrier in humans—the blood–brain barrier—but can also progressively accumulate in brain tissues, posing potential long-term health risks. Exposure to NPs has been shown to induce neuroinflammation and oxidative stress, therefore posing a potential threat to central nervous system health [9]. Exposure during pregnancy represents a particularly sensitive context for NP toxicity. Increasing evidence indicates that NPs can cross the placental barrier, resulting in the abnormal development of embryos, fetuses, and placentas, and leading to adverse outcomes such as neurodevelopmental abnormalities, behavioral disorders, and reproductive system damage in offspring [10,11,12]. Animal studies further resemble these observations: female mice exposed to 50 nm NPs during pregnancy and lactation exhibited detectable accumulation of NPs in the brains of their offspring, which was associated with impaired brain development and function [13]. Similarly, NPs have been shown to penetrate and accumulate in the developing embryos of zebrafish [14]. Collectively, these findings indicate that NPs can enter the brain either by directly crossing the blood–brain barrier or through the maternal–fetal pathway, ultimately inducing neurotoxic effects.

**Figure 1:**
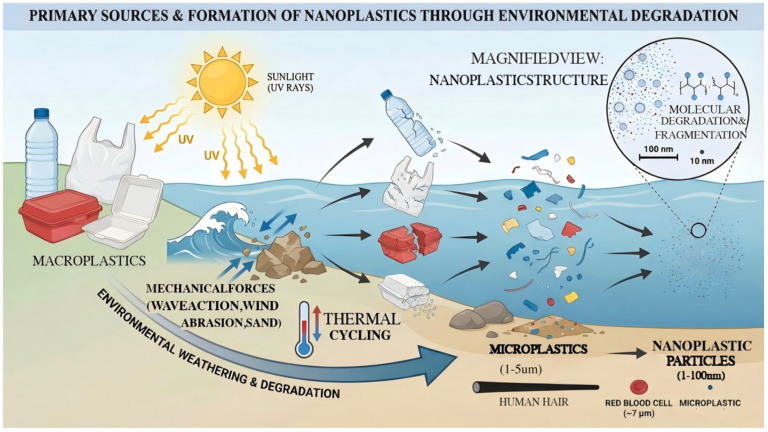
Sources of NPs. The primary sources of nanoplastic particles encompass a wide range of plastic products commonly used in daily life. Under natural environmental conditions, these products gradually break down and decompose into nanoscale plastic particles through long-term natural wear processes such as weathering, mechanical friction, as well as aging mechanisms, including ultraviolet radiation and oxidation reactions. Image created with BioRender.com. Wan, F. (https://BioRender.com/8hfb87e) is licensed under CC BY 4.0.

Here, we summarize recent advances in understanding the neurobehavioral abnormalities and the potential mechanisms induced by NPs. We specifically focus on their effects on the nervous system through oxidative stress and inflammation, ferroptosis, cellular autophagy, and the gut–brain axis (Figure 1).

## 2. Data Sources

This review was designed as a targeted, non-systematic narrative review of the neurotoxic effects of NPs, with an emphasis on experimental evidence related to neurotoxicity, mechanistic pathways, and the potential relevance of these findings to neurodegenerative disease. The literature retrieval was conducted with PubMed as the primary database, supplemented by Google Scholar and manual screening of the reference lists of relevant review articles and key original studies.

The search was last updated on 16 April 2026. We considered studies published between 1 January 2000 and 31 December 2025. The PubMed search strategy was: ((nanoplastic*[Title/Abstract]) OR (nano-plastic*[Title/Abstract])) AND ((neurotox*[Title/Abstract]) OR (brain[Title/Abstract]) OR (nervous system[Title/Abstract]) OR (neuron*[Title/Abstract]) OR (neurodegenerat*[Title/Abstract]) OR (Alzheimer*[Title/Abstract]) OR (Parkinson*[Title/Abstract]) OR (Huntington*[Title/Abstract]) OR (amyotrophic lateral sclerosis[Title/Abstract]) OR (ALS[Title/Abstract])) AND (“1 January 2000”[Date-Publication]: “31 December 2025”[Date-Publication]).

For supplementary searching in Google Scholar, we used combinations of the following terms: “nanoplastics neurotoxicity”, “nanoplastics brain”, “nanoplastics neurodegenerative disease”, “nanoplastics Alzheimer”, “nanoplastics Parkinson”, and “nanoplastics oxidative stress neuron”. To improve reproducibility, only the first 200 results sorted by relevance were screened in Google Scholar. Additional articles were identified through backward citation tracking of eligible articles and recent reviews.

Eligibility criteria were predefined. We included peer-reviewed original studies and relevant reviews that addressed at least one of the following topics: (1) neurotoxicity of nanoplastics in in vitro or in vivo models; (2) mechanisms potentially linking NPs exposure to nervous system injury, including oxidative stress, neuroinflammation, ferroptosis, mitochondrial dysfunction, proteostasis imbalance, gut–brain axis dysregulation, or blood–brain barrier disruption; and (3) evidence discussing the possible relevance of NPs exposure to neurodegenerative diseases, including Alzheimer’s disease, Parkinson’s disease, amyotrophic lateral sclerosis, and Huntington’s disease. We excluded articles that focused exclusively on microplastics without nanoscale characterization, did not address nervous system outcomes, lacked sufficient methodological information, were conference abstracts or non-peer-reviewed publications, or were not available in full text.

The screening process was performed in three steps. First, records retrieved from PubMed and supplementary sources were merged and duplicates were removed. Second, titles and abstracts were screened for apparent relevance to NPs-associated neurotoxicity. Third, full texts were assessed for eligibility according to the predefined inclusion and exclusion criteria. The literature flow was as follows: 413 records were identified through PubMed and 68 additional records were identified through Google Scholar screening and manual reference searching, yielding 481 total records. After the removal of 91 duplicates, 390 records were screened by title and abstract. Of these, 248 were excluded for lack of direct relevance. The remaining 142 articles underwent full-text review; 41 were excluded because they did not specifically address nanoplastics and neurotoxicity, lacked sufficient methodological detail, or did not provide mechanistic or disease-relevant information. Ultimately, 101 articles were included in the qualitative synthesis.

Because this manuscript is a narrative review rather than a formal systematic review or meta-analysis, we did not apply a standardized risk-of-bias tool. However, the methodological quality of the included studies was appraised qualitatively according to the following aspects: clarity of NP characterization (polymer type, particle size, surface properties, and aging status), exposure design (dose, duration, and route), appropriateness of experimental models, rigor of outcome assessment, and transparency of statistical reporting. When interpreting the evidence, greater weight was given to studies with well-characterized nanoplastic materials, biologically relevant exposure paradigms, and clearly reported neurotoxic endpoints. At the same time, we explicitly considered inconsistencies across models and the limited direct human evidence when discussing the translational relevance of the findings.

Table. Reproducible search framework used in this review

Item: Review type

Details: Targeted non-systematic narrative review

Item: Databases

Details: PubMed (primary); Google Scholar and reference-list screening (supplementary)

Item: Search period

Details: 1 January 2000 to 31 December 2025

Item: Last search date

Details: 16 April 2026

Item: PubMed search string

Details: ((nanoplastic*[Title/Abstract]) OR (nano-plastic*[Title/Abstract])) AND ((neurotox*[Title/Abstract]) OR (brain[Title/Abstract]) OR (nervous system[Title/Abstract]) OR (neuron*[Title/Abstract]) OR (neurodegenerat*[Title/Abstract]) OR (Alzheimer*[Title/Abstract]) OR (Parkinson*[Title/Abstract]) OR (Huntington*[Title/Abstract]) OR (amyotrophic lateral sclerosis[Title/Abstract]) OR (ALS[Title/Abstract])) AND (“1 January 2000”[Date-Publication]: “31 December 2025”[Date-Publication])

Item: Google Scholar approach

Details: Supplementary search using predefined keyword combinations; first 200 results screened by relevance

Item: Records identified

Details: PubMed, n = 413; supplementary sources, n = 68; total, n = 481

Item: Final inclusion

Details: 101 articles included in qualitative synthesis

Literature screening flow

Records identified from PubMed: n = 413

Additional records from Google Scholar and manual reference screening: n = 68

Total records: n = 481

Duplicates removed: n = 91

Records screened by title/abstract: n = 390

Records excluded after title/abstract screening: n = 248

Full-text articles assessed: n = 142

Full-text articles excluded: n = 41

Final studies included in qualitative synthesis: n = 101

## 3. Factors Influencing the Neurotoxicity of NPs

Studies have shown that the toxicity of nanoparticles (NPs) is influenced by multiple material characteristic factors, encompassing various aspects such as the type of NPs, particle size, surface area, and surface modifications.

### 3.1. Polymer Type

It is particularly noteworthy that there are significant differences in the neurotoxic mechanisms induced by different types of NPs. The chemical composition of nanoplastics is distinctly characterized by their polymer types; variations in polymer types exert a substantial and non-trivial influence on the neurotoxicity of nanoplastics. Within the current research paradigm, polystyrene (PS) nanoplastics have emerged as the focal point in neurotoxicity research, attributable to their well-established preparation methodologies and relatively homogeneous properties. A plethora of studies have been undertaken on PS nanoplastics, resulting in notable advancements. Nevertheless, preliminary research findings suggest that nanoplastics composed of different polymers exhibit significant disparities in toxicological manifestations. This discrepancy likely arises from the distinct physicochemical properties inherent to different polymers, encompassing their unique molecular architectures, surface charge distributions, and hydrophobicity profiles, which, in turn, modulate their biological interactions, including interactions with neural cell membranes, intracellular uptake and distribution patterns, as well as the regulation of intracellular signaling pathways [15]. Relevant studies have revealed significant differences in the toxicity exhibited by five distinct types of nanoplastics (namely PE-NPs, PET-NPs, PMMA-NPs, PP-NPs, and PS-NPs) on SH-SY5Y cells. Specifically, PE-NPs and PP-NPs demonstrated stronger cytotoxicity, while PS-NPs showed relatively weaker toxicity; PET-NPs and PMMA-NPs exhibited moderate toxic characteristics. These differences may be closely related to factors such as the hydrophobicity of nanoplastics, surface properties, and internalization efficiency. Among them, PE and PP, due to their strong hydrophobicity, are more prone to bind with cell membranes and enter the intracellular space, subsequently inducing cellular damage through mechanisms such as oxidative stress [16].

### 3.2. Size

Previous studies have shown that NPs have a broader distribution and more severe tissue accumulation compared to MPs, being detectable even in deeper organs such as the testes and kidneys. These findings suggest that the smaller particle sizes facilitate penetration across biological barriers and their systemic distribution [17]. Consequently, smaller NPs (100 nm or 500 nm) exhibit stronger toxicity in organs protected by biological barriers such as developing embryos and the nervous system [18].

### 3.3. Surface Modification

In addition to particle size, the surface chemical properties have an important role in the uptake and toxicity of NPs. Studies has found that 100 nm polystyrene particles with different surface charges exhibit distinct cellular uptake features. For example, in liver cells, 100 nm neutral particles show the highest uptake efficiency, while in intestinal cells, 100 nm amino-modified PS particles with positive charges are able to be taken up more efficiently than other particles [19]. Notably, cholesterol-modified nanoplastics demonstrate a higher propensity to penetrate the blood–brain barrier compared to protein-modified nanoplastics, a phenomenon that may be closely associated with cholesterol-mediated transmembrane transport mechanisms [20]. In summary, as a critical factor, surface modification directly influences the accumulation process of nanoplastics in the central nervous system and the generation of toxic effects by regulating their cellular uptake efficiency, altering subcellular localization, and affecting the activation status of signaling pathways. Consequently, it serves as a key parameter for assessing the neurotoxic risk of nanoplastics. Furthermore, numerous studies have reported that co-exposure to NPs with environmental pollutants, such as organic substances and heavy metals, may enhance neurotoxic effects. On the one hand, NPs can adsorb harmful substances from surrounding environment through physical and chemical interactions, thereby exacerbating the toxic effects of these substances. On the other hand, surfaces with lipophilic pollutants may facilitate the penetration of NPs through biological membranes, promoting their accumulation and damage within the body [17,21].

## 4. Neurotoxic Effects of NPs

### 4.1. Neurobehavioral Changes Induced by NPs

A growing body of evidence indicates that NPs can adversely affect learning, memory, and other neurobehavioral functions in different animal models, including zebrafish and rodents. Kyu-Seok et al. reported that exposure to NPs can alter color preference behavior in zebrafish, which is related to their learning and memory processes. Using a T-maze exploration task, the authors demonstrated that both sexes of zebrafish exposed to NPs showed a significantly prolonged latency to enter the reward area for the first time compared with the control group. Moreover, after 4 days of reward learning, the cumulative time spent in the reward area in NPs-exposed zebrafish group was markedly reduced compared to that of control group, indicating impaired learning acquisition and memory retention [22,23]. Consistent neurobehavioral changes have also been observed in the early embryonic developmental stage. Mónica Torres-Ruiz et al. found that exposure to NPs affected tail-coil activity in zebrafish embryos at 24 h post fertilization (hpf), characterized by an increased burst count but a decreased movement duration. A light–dark movement response test at 120 hpf revealed a biphasic behavioral pattern (initial hyperactivity followed by hypoactivity) induced by different exposure concentrations of NPs. These findings indicate that exposure to NPs disrupt neuro-motor regulation during early embryonic development, which may potentially be linked to cognitive dysfunction [24].

Numerous studies have demonstrated that NPs can accumulate in the brains of mice and induce impairments in emotional regulation and cognitive functions. In an open field test, Prosperi et al. observed a NPs dose-dependent decrease in the distance traveled, time spent in central area activity, and frequency of center entries, suggesting that NPs induced a suppressed exploratory behavior, which is associated with cognitive dysfunction [25]. Using the Morris water maze, Li et al. reported that NPs-exposed mice required more time to locate the hidden platform, had fewer crossings of the target platform area, and spent less time in the target quadrant, indicating deficits of spatial learning and memory [26]. In a novel object recognition test, Sun et al. observed that NPs-exposed mice showed significantly lower novelty recognition indices, indicating an impairment in short-term recognition memory [27]. Additionally, sensory systems can also be affected. Prosperi et al. showed that 30-day exposure to NPs resulted in persistent olfactory discrimination deficits, suggesting long-term damage to olfactory sensitivity and recognition [25]. Collectively, these findings indicate that NPs exposure can induce a broad spectrum of neurobehavioral impairments, with cognitive deficits including memory, learning, motor skills, and olfactory functions [25,27].

It is noteworthy that, although numerous studies have demonstrated neurobehavioral impairments in animal models following NPs exposure, several studies have failed to detect noticeable neurobehavioral changes under certain experimental conditions. The discrepancies are likely attributable to differences in NPs exposure concentrations or duration, and experimental design. For instance, Xing et al. reported that exposure alone to 100 ng/mL of NPs had no significant adverse effect on the locomotor activity of embryos/larvae in zebrafish. However, when combined with exposure to ammonia (NH_3_), the neurotoxicity of NPs was significantly enhanced in the embryos of zebrafish, leading to a pronounced reduction in motor activity [28]. These findings suggests that NPs may exhibit limited neurotoxicity under single exposure conditions but can have enhanced neurotoxic effects under co-exposure conditions [28]. The failure to observe significant behavioral changes may also be attributed to differences in animal models or developmental stages. Embryonic and larval stages are generally more sensitive to NPs exposure than adult stages; neurobehavioral changes in adult zebrafish are relatively weak or less often reported. Sarasamma et al. reported that chronic high concentration exposure to 70 nm PS NPs induced significant behavioral changes in multiple endpoints (swimming, aggression, shoaling, predator avoidance, circadian rhythm, etc.) in adult fish; meanwhile, changes in neurotoxicity and oxidative stress-related biomarkers were also observed. NPs were detectable and enriched in tissues under these exposure conditions [29]. In contrast, short-term, low-concentration (e.g.,100 ng/mL) exposure alone to NPs did not alter larval locomotor activity in embryos or larvae unless NPs exposure was combined with other environmental pollutants such as ammonia [28,29]. Overall, these findings suggest that what causes the neurobehavioral changes induced by NPs exposure to be detectable is determined by various exposure conditions and selection of animal models (Table 1).

Beyond exposure conditions, the physicochemical properties of nanoplastics critically affect their neurotoxic potential. Particles size is a particular key characteristic, as smaller nanoplastics (<100 nm) exhibit stronger penetrability. Studies have shown that the toxicity of nanoplastics is enhanced after mimicking environmental aging processes such as photodegradation [30]. Li et al. simulated environmental light exposure to induce functional group modification and morphological changes on the NPs surface. The results revealed that aging NPs caused significantly greater locomotor inhibition and motor-neuron developmental damage than non-aged NPs under the same concentrations, indicating that environmental aging processes are one of the key factors enhancing NPs toxicity. It may partly explain why NPs derived from the natural environment generally exhibit greater toxicity than pristine commercial particles.

Previous studies have also demonstrated that exposure to 20 nm NPs induces more severe neuroinflammation and neurobehavioral abnormalities compared with larger NPs (≥100 nm) [31]. Using C14-labeled PS NPs, Wu et al. [31]. demonstrated that 20 nm nanoparticles accumulated at higher levels in the brain than 100 nm nanoparticles, sequentially activating neuroinflammation. Moreover, the authors proved that NPs could directly bind to the TLR4/MD-2 complex and furtherly induce conformational changes through the analysis of molecular dynamics simulations. The 20 nm NPs exhibited a stronger capacity to bind and induce dimerization of this complex, providing direct mechanistic evidence linking nanoparticle size to neurotoxicity intensity. Thus, studies employing larger NPs (≥100 nm) or failing to accurately quantify brain accumulation may underestimate the neurobehavioral impairments induced by NPs.

**Table 1:** Summary of neurobehavioral alterations induced by NPs.

Authors (Year)	Particle Size	Polymer Type	Exposure Concentration	Exposure Days	Experimental Model (Species, Stage)	Main Outcomes
Sun et al. [27] 2024	80 nm	Polystyrene	2.5 mg/kg, 5 mg/kg, 10 mg/kg	7 days and 28 days	C57BL/6 J male mice	Impaired exploratory ability and deficits in spatial learning and memory
Kaur et al. [32] 2024	2 µm	Polystyrene	0.1 mg/mL and 1 mg/mL	15 days, 30 days, 60 days	Male Swiss albino mice	Impaired exploratory behavior and declined spatial learning and memory abilities.
Yang et al. [33] 2022	0.1 µm, 1 µm	Polystyrene	1 mg/day	17 days	Pregnant C57BL mice	Anxiety-like behavior.
Jin et al. [34] 2022	0.5 µm, 4 µm, 10 µm	Polystyrene	100 ng/mL and 1000 ng/mL	180 days	Six-week-old male BALB/c mice	Impaired spatial learning and memory, decreased short-term recognition memory, altered behavioral patterns.
Chen et al. [35] 2024	80 nm, 200 nm, 500 nm	Polystyrene	0.1, 0.5, 1, 5, 10, 25 and 50 mg/L	96 h hatch	Zebrafish embryo	The swimming distance of larvae decreased under 80 nm PS exposure, while increased under 200 nm and 500 nm conditions
Torres-Ruiz et al. [24] 2023	30 nm	Polystyrene	0.1 mg/L, 0.5 mg/L and 3 mg/L	120 h post-fertilization	Zebrafish embryos and larvae	Increased tail curling activity, altered swimming behavior, anxiety-like behavioral changes, startle response, and impaired habituation
Li et al. [30] 2024	1.07 ± 0.04 µm, 0.96 ± 0.07 µm	Photoaged polystyrene	0.1 µg/L, 1 µg/L, 10 µg/L, 100 µg/L	120 h (approximately 5 days)	Wild-type zebrafish (AB strain) Transgenic zebrafish strain Tg(hb9-GFP)	Decreased average swimming speed and reduced movement distance
Zhou et al. [18] 2023	100 nm, 500 nm, 1000 nm	Polystyrene	10 mg/L	5 days	Non-transgenic zebrafish and two transgenic zebrafish strains: Tg(HuC-GFP) and Tg(Hb9-GFP)	Decreased average speed and movement distance, inhibition of locomotor activity.
Oger et al. [36] 2024	5 µm, 250 nm	Polystyrene	1000 µg/L	96 h (approximately 4 days)	Zebrafish embryos and larvae	The visual motor response (VMR) and vibratory startle response (VSR) were negatively affected
Kashiwada [37] 2006	40 nm	Polystyrene	10 mg/L	In medium, for 7 days	Japanese rice fish (*Oryzias latipes*)	Decreased swimming activity, diminished escape response to external stimuli, and the appearance of postural instability and abnormal swimming trajectories
Chen et al. [38] 2017	50 nm	Polystyrene	1 mg/L, with or without 0.78 and 1.0 µg/L BPA	In water, for 3 days	Zebrafish, larvae (*Danio rerio*)	Decreased locomotor activity, increased anxiety-like behavior, delayed response, and abnormal movement patterns
Hua et al. [39] 2024	100 nm	Polystyrene	0.1 ng/mL, 1 ng/mL and 10 ng/mL	4.5 days	*Caenorhabditis elegans*	Reduced head bobbing and body bending

### 4.2. Pathological Changes

Exposure to NPs induces neurotoxicity by triggering neuropathological changes in normal neural tissues. A significant amount of evidence indicates that NPs can cross and disrupt critical biological membrane barriers, thereby facilitating nanoparticle penetration into the neuronal system. Hua et al. demonstrated that NPs can penetrate the blood–brain barrier (BBB) and damage its structural integrity [39]. Consistently, Vethaak et al. reported that NPs were endocytosed by the vascular endothelial cells of BBB, leading to the disruption of tight junction complexes and increased BBB permeability, which are further worsened by NPs-induced cytokine release, which promotes endothelial cell injury [39,40]. In addition to crossing the BBB, NPs can traverse the placental barrier during mice pregnancy, enter the fetal brain, and accumulate in multiple brain regions (e.g., cerebellum, hippocampus, striatum, and prefrontal cortex) [13]. Such accumulation in the fetal brain results in neurodevelopmental defects and brain morphological changes in offspring. Manjyot et al. reported that NPs-exposed offspring mice exhibited dystrophic changes in the cerebral cortex, featured by shrunken and irregular cell nuclei, distorted neuronal morphology, and hyperchromatic nuclei. Additionally, in the hippocampus, pronounced neuropathological alterations were observed, including increased vacuolation, vascular dilation, reduced thickness of the pyramidal layer, and a significant reduction in pyramidal neuron numbers in the CA1, CA2, and CA3 regions [41]. A loss of pyramidal neurons may lead to a decreased number of synapses and impairment of synaptic transmission efficiency, thereby compromising brain functions.

In addition to neuronal loss, exposure to NPs has been shown to impair myelin development in the cerebellar regions of offspring mice. Prenatal exposure to NPs reduced the expression of myelin-related proteins, myelin basic protein (MBP) and myelin oligodendrocyte glycoprotein (MOG), suggesting that NPs exposure may alter these proteins’ composition and structural integrity. Electron microscopy analysis revealed that the g ratio (a measure of myelination) significantly increased in the NPs exposure offspring, indicating impaired myelination. These findings demonstrate that prenatal NPs exposure adversely affects cerebellar myelin development [42]. Additionally, NPs can be phagocytosed by immune cells and form cellular microemboli, which subsequently occlude cortical capillaries in the brain, leading to inadequate local cerebral blood flow perfusion. This chronic impairment of blood flow can induce cerebral ischemia and hypoxia, ultimately exacerbating the progression of neurological damage [43]. Furthermore, the nanoplastic-exposed group exhibited an increase in arterial plaque area, accompanied by significantly elevated levels of pro-inflammatory metabolites (such as prostaglandin E2). This not only led to reduced stability of the vascular wall but also substantially increased the risk of thrombosis, thereby indirectly exacerbating the risk of stroke [44].

In summary, in vivo and in vitro studies consistently demonstrated that exposure to NPs disrupt BBB integrity by destroying tight junction complexes and increasing barrier permeability, crossing the placenta barrier to enter into the developing fetal brain, and accumulating in vulnerable regions such as the cerebellum and hippocampus, thereby triggering a cascade neuropathological changes, including neuroinflammation, neuronal apoptosis, synaptic dysfunction, and impaired myelin development [45,46,47].

## 5. Potential Mechanisms Underlying NPs Induced Neurotoxicity

To date, many studies have indicated that exposure to NPs can induce neurotoxic effects through multiple mechanisms, including neuroinflammation, oxidative stress, ferroptosis, dysregulated autophagy, and disruption of the gut–brain axis. Among these potential interrelated mechanisms, oxidative stress is a central and early molecular event that may link NPs exposure to subsequent neuropathological outcomes. The following sections summarize the current main understanding of these pathways.

### 5.1. Oxidative Stress

Oxidative stress refers to a pathological state characterized by an imbalance between oxidation and anti-oxidation, generally in excessive oxidative activity within cells and tissues [48]. Oxidative stress is a result of oxidative intermediates accumulation within cells and is recognized as a key factor in aging and various diseases [49,50]. At the cellular level, oxidative stress arises from disrupted cellular redox homeostasis and the excessive generation of reactive oxygen species (ROS) [51], which can damage lipids, proteins, and nucleic acids, ultimately leading to mitochondrial dysfunction even cell death [52].

**Figure 2:**
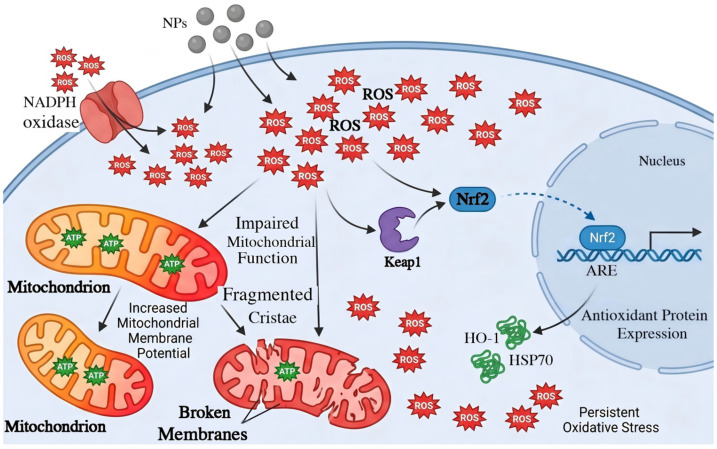
Nanoplastics induce excessive accumulation of reactive oxygen species (ROS) and impair ATP synthesis by activating NADPH oxidase and damaging the mitochondrial electron transport chain. Although this process can activate the Nrf2-Keap1 antioxidant defense pathway and induce the expression of downstream protective genes, under conditions of long-term exposure, the redox homeostasis remains unrecoverable, ultimately leading to persistent mitochondrial dysfunction and neuronal damage. Image created with BioRender.com. Wan, F. (https://BioRender.com/f67k63g) is licensed under CC BY 4.0.

Many current studies suggest that oxidative stress plays a critical role in neurotoxicity induced by NPs. Exposure to NPs has been shown to markedly elevate intracellular ROS levels and activate NADPH oxidase, resulting in an increase in mitochondrial membrane potential and a decrease in ATP synthesis, thereby impairing mitochondrial function [53]. Excessive ROS can further activate the nuclear factor erythroid 2-related factor 2 (Nrf2) signaling pathways. Under oxidative stress, Keap1 dissociates from Nrf2, enabling Nrf2 to translocate into the nucleus and induce the transcription of downstream antioxidant response-related genes, thereby mitigating redox imbalance. Research has demonstrated that exposure to NPs inhibits the expression of Keap1 while enhancing the expression of Nrf2, consequently leading to the expression of antioxidant proteins such as heme oxygenase-1 and heat shock protein 70, and activating the cellular antioxidant system. However, despite activation of the Nrf2 pathway, the intracellular ROS level does not return to normal in the context of long-term exposure to NPs, ultimately leading to persistent mitochondrial dysfunction [54,55]. It is noteworthy that antioxidants (with N-acetylcysteine as a typical representative) demonstrate potent effects by significantly reducing nanoplastics-induced reactive oxygen species (ROS) levels, lipid peroxidation, and apoptotic rates [56]. This groundbreaking discovery opens new avenues for antioxidant-based interventions against nanoplastics neurotoxicity and provides crucial experimental evidence (Figure 2).

In addition, an increased ROS level is involved in many pathological processes, including cellular senescence. Although direct evidence of senescence induced by NPs exposure in neural cells is limited, some studies have shown that exposure to NPs leads to a dose-dependent burst of ROS in ATCC-derived mouse spermatogonial cells [57]. ROS overproduction is associated with the activation of Sirt1 and a DNA damage response. Moreover, treatment with the ROS inhibitor N-acetyl-L-cysteine (NAC) restored the cell cycle progression and reduced the proportion of β-galactosidase positive cells, a hallmark of cellular senescence [58]. These findings may suggest that increased ROS levels induced by NPs exposure also contribute to senescence in neural cells.

The study has revealed that oxidative stress induced by NPs exposure not only activates the Keap1/Nrf2 signaling pathway, which triggers inflammatory responses and mitochondrial damage, but also that excessive ROS can activate the intracellular ERK-MAPK pathway, thereby promoting neurotoxicity [59,60,61]. Specifically, exposure to NPs leads to copper accumulation in the mouse brain, which sequentially elevates ROS levels, accompanied by the suppression of endogenous antioxidant defenses. Oxidative stress activates the ERK-MAPK pathway via phosphorylation, which in turn promotes copper accumulation and the expression of copper death-related proteins. In this process, oxidative stress acts both as an initiator of copper-induced cell death and as an amplifier of this process through the activation of the MAPK pathway [62]. Meanwhile, sustained activation of the ERK-MAPK pathway further inhibits mitochondrial autophagy, preventing the clearance of damaged mitochondria. Eventually, the accumulation of dysfunctional mitochondria in cells exacerbates energy metabolism disorders and increases the susceptibility of neuronal cell death [63]. This cascade of interrelated molecular events is particularly pronounced in neuronal cells, characterized as synaptic dysfunction and neurodegenerative changes [64]. Moreover, the accumulation of copper and ROS overproduction in the cell form a positive feedback loop, in which elevated copper levels promote ROS production, and increased ROS further enhance the accumulation of copper. This reinforcing cycle continuously intensifies oxidative stress, ultimately driving neurons toward irreversible copper-dependent cell death program and neuronal damage [65].

In addition to directly dysregulating signaling pathways, NPs exposure-induced oxidative stress can significantly disrupt neurotransmitter homeostasis and thereby induce neurotoxicity. Faezeh et al. reported that ROS derived from oxidative stress induced by NPs exposure can directly oxidize neurotransmitters, such as acetylcholine and glutamate, destroying their molecular structure and biological activity, causing imbalance in synaptic neurotransmitter levels. Furthermore, NPs exposure also inhibits the activity of neurotransmitter-synthesizing enzymes, leading to abnormal accumulations of acetylcholine (Ach) in the synaptic cleft, and blocking the conversion of glutamate to glutamine. These alterations result in excessive accumulation of acetylcholine (Ach) and glutamate in the synaptic cleft and extracellular space, triggering hyperexcitation or synaptic desensitization. Meanwhile, with the reduction in the activity of α-ketoglutarate dehydrogenase (α-KGDH), the tricarboxylic acid cycle is suppressed, resulting in insufficient ATP production and impaired energy supply for neurotransmitter synthesis and transport [66].

Some studies have shown that oxidative stress induced by NPs exposure can cause DNA damage in human neural stem cells. Although antioxidant genes, such as Cu/ZnSOD1 and CAT, are activated following NPs exposure, these responses are not sufficient to completely eliminate ROS. Excessive ROS can directly attack DNA molecules, causing base oxidation and strand breaks. Moreover, one study revealed that exposure to NPs disrupted the DNA repair system, ultimately leading to the genomic instability of neuronal cells. These processes may be closely related to neurodevelopmental toxicity and increase the risk of neurodegenerative diseases [67].

### 5.2. Neuroinflammation

Neuroinflammation is one of the most crucial pathological mechanisms underlying neurological diseases [68,69,70]. It can serve not only as a direct trigger for certain neurological disorders but also as a consequence of neurological diseases, thereby exacerbating disease progression. Environmental factors are recognized as one of the initiators of neuroinflammation; NPs, as emerging environmental pollutants, have been shown to induce neuroinflammatory responses [71,72].

Numerous studies have demonstrated that exposure to NPs, eventually leads to neuronal damage and loss. Once NPs span across the BBB and enter the brain, they are initially recognized and phagocytosed by resident immune cells of the central nervous system, particularly microglia. This process promotes microglia polarization from a resting state to the M1 phenotype. It is well established that M1-type microglia secrete a wide range of pro-inflammatory cytokines and cytotoxic factors, including IL-1α, TNF-α, C1q, HMGB1, IL-6, and IL-17. These pro-inflammatory factors collectively contribute to NPs exposure-induced neuroinflammation. Among them, IL-6 acts as a major inducer of acute-phase protein synthesis, while IL-17 activates the NF-κB and MAPK signaling pathways. Activation of the NF-κB signaling cascade promotes the expression of pro-inflammatory cytokines, chemokines, and inducible enzymes, such as iNOS and COX-2, thereby triggering and amplifying neuroinflammation [73]. Additionally, the synergistic effects of IL-1α, TNF-α, and C1q make the transformation of quiescent astrocytes into neurotoxic A1 reactive astrocytes, which are featured by elevating the expression of complement C3. Meanwhile, these A1-type astrocytes further aggravate microglial activation through secreting chemokines, forming a positive feedback loop that intensifies the inflammatory response [74].

**Figure 3:**
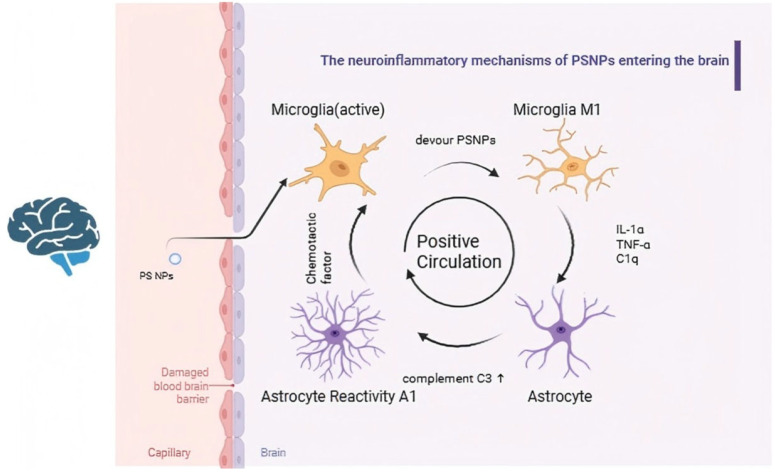
Neuroinflammatory mechanisms induced by NPs exposure. After spanning across the BBB, NPs activate microglia and astrocytes, triggering cytokine release and complement activation. These processes form a positive feedback cycle that sustains neuroinflammation and subsequent neuronal damage. Image created with BioRender.com. Wan, F. (https://BioRender.com/o83b0ll) is licensed under CC BY 4.0.

In addition to directly influencing immune cell activation, exposure to NPs can induce neuroinflammation by activating the complement system as well. Under physiological conditions, complement C1q selectively deposits on synapses during neurodevelopment; sequentially, microglia eliminate excess synapses via interactions with C3a receptors. However, exposure to NPs aberrantly activates the complement cascade in the brain, leading to non-specific labeling of both healthy and damaged synapses. This abnormal activation enhances microglial phagocytic activity, leading to excessive synaptic elimination and subsequent neuronal degeneration. Degenerated neurons further activate microglia and astrocytes, thereby establishing a self-perpetuating inflammatory cycle reliant on the overactivation of complement cascades [74] (Figure 3).

NPs exposure can cause neuroinflammation by inducing intracellular signaling alterations. Studies have reported that exposure to NPs causes cellular membrane stress and lipid bilayer disruption, potentially leading to transient membrane permeabilization and the release of damage-associated molecular patterns (DAMPs). Notably, HMGB1 is one of the key DAMP molecules, which interacts with toll-like receptors and receptors for advanced glycation end products as long as these are being released into the extracellular space. This interaction activates the MAPK pathway, ultimately coordinating the production of inflammatory cytokines and extracellular matrix proteins, and upregulating the expression of iNOS [75]. Therefore, NPs exposure induces neuroinflammation through dual mechanisms involving complement system overactivation and the DAMPs-MAPK signaling pathway, leading to excessive synaptic pruning, neuronal degeneration, and sustained release of inflammatory factors, thereby forming a vicious cycle of neuroinflammatory damage.

Many current studies have clearly demonstrated that exposure to NPs induces polarization of macrophages to the pro-inflammatory M1 phenotype while inhibiting anti-inflammatory M2-type polarization, resulting in an imbalance in macrophage phenotypes. Such a polarization imbalance may exacerbate neuroinflammatory progression through various complex biological mechanisms and significantly impede neural functional recovery [76].

Overactivation of M1-type macrophages contributes to neuronal damage, accompanied by secreting various neurotoxic mediators, including pro-inflammatory cytokines (e.g., IL-1β, TNF-α, and IL-12), oxidative stress related factors (such as iNOS and NADPH oxidase), and proteolytic enzymes such as MMPs, all of which can damage neuronal cells [77]. Moreover, activation of M1-type macrophages increases BBB permeability, facilitating the infiltration of peripheral immune cells and exacerbating central nervous system damage. Sustained oxidative imbalance can maintains macrophages in a persistently activated state, which in turn promotes immune cell recruitment and amplifying the inflammatory response and ultimately leads to chronic neuroinflammation. Additionally, studies have shown that M1-derived proteases degrade extracellular matrix components and disrupt tissue architecture, thereby inhibiting axonal regeneration and neural repair [78,79].

In contrast, M2-type macrophages play a key role in neuroprotection and neuron repair. They secrete a variety of neurotrophic factors, including epidermal growth factor, vascular endothelial growth factor, and transforming growth factor-β, as well as anti-inflammatory factors such as IL-10, TGF-β, and IL-13. These all factors function together to form a protective microenvironment for neuronal survival and regeneration. Furthermore, M2 macrophages exhibit strong phagocytic capacity, efficiently clearing apoptotic cells and cellular debris within the inflammatory response area, which helps to limit secondary inflammatory damage [80]. Importantly, the anti-inflammatory factors released from M2-type macrophages can effectively inhibit the overactivation of pro-inflammatory immune cells, thereby playing a critical role in maintaining the neuroimmune homeostasis [81]. Given the above functional properties, therapeutic strategies aimed at promoting macrophage polarization to the M2 phenotype after nerve injury may be a promising way to promote the recovery of neural function [82,83].

### 5.3. Ferroptosis

Ferroptosis is a novel form of iron-dependent programmed cell death that is distinct from other types of cell death such as apoptosis, necrosis, and autophagy. Its defining feature is the lethal accumulation of lipid peroxides caused by iron buildup within cells, which ultimately leads to cell membrane rupture and cell death. Many studies have revealed that ferroptosis is closely associated with various neurodegenerative diseases, as abnormal iron accumulation has been observed in specific brain regions of patients with neurodegenerative diseases [84]. Recent studies further reveal that exposure to NPs can trigger ferroptosis and subsequently induce neurotoxicity in neuronal and glial cells.

Research has shown that exposure to NPs induces oxidative stress and activates inflammasomes to release inflammatory cytokines accompanied with particles penetrating into microglial cells in mouse brains, while simultaneously disrupting iron and lipid metabolism. Elevated ROS initiates inflammatory responses and ferroptosis via activating the FTH1 signaling pathway, thereby exerting neurotoxic effects [85]. In addition, in vivo studies have demonstrated that the exposure of pregnant SD rats to NPs significantly increases ROS levels in the hippocampus region of their offspring brains. Elevated ROS activates the P53 signaling pathway, which subsequently induces ferritinophagy through NCOA4. In the NPs-exposed group, the expression levels of NCOA4 and the autophagy-related protein LC3II were markedly elevated, whereas FTH1 expression was significantly reduced. As FTH1 binds to NCOA4 and mediates ferritinophagy, a reduction in FTH1 indicates that NPs exposure may induce enhanced ferritin degradation and excessive iron release. The released iron ions participate in Fenton reactions, promoting lipid peroxidation and activating ALOX15, ultimately triggering ferroptosis in hippocampal neurons [86].

In addition to directly inducing ferroptosis in neural cells, NPs exposure may also exert neurotoxicity through immunogenic cell death, mediated by ferroptosis. Although it remains unclear whether this process directly leads to neuronal damage, studies have shown that NPs can be internalized by mouse intestinal epithelial cells via endocytosis, resulting in increased intracellular ROS levels and p53 activation, which sequentially suppresses Slc7a11 expression, thereby promoting ferroptosis. Ferroptosis induced by NPs exposure in mouse colon tissue facilitates the release of antigens, which are subsequently recognized by dendritic cells and presented to T cells. This process enhances CD8+ T cell recruitment and initiates immunogenic cell death, eventually altering the immune microenvironment, and inducing cytotoxic responses [87]. Moreover, studies have demonstrated that ferroptosis induced by NPs exposure leads to intracellular environmental disturbances and cellular damage, resulting in cellular senescence. Therefore, based on current findings and understandings, ferroptosis may be associated with the development of cellular senescence through activating aging-related signaling pathways, particularly the p53 signaling pathway [88].

The study has shown that the inhibition of ferroptosis using Fer-1 or DFP can alleviate the aging of mouse placentas and human syncytiotrophoblast (STB) cells induced by NPs exposure. This alleviative effect is evidenced by a reduction in the number of SA-β-gal positive cells and downregulation of the expression of the senescence-associated proteins p21 and γH2AX [89]. Although current studies cannot conclusively confirm whether NPs exposure induced neuronal senescence directly contributes to neurotoxicity, it is clear that lipid peroxides accumulation during ferroptosis can disrupt cellular membrane structure and impair cellular metabolism and signaling.

The neurotoxic mechanisms of NPs induced ferroptosis are diverse. In addition to directly causing neuronal damage, ferroptosis may also promote neuronal injury and dysfunction through a series of complex systemic pathways, including the liver–brain axis and lung–brain axis. The disruption of systemic metabolism enables excessive glutamine and glutamate to span across the BBB and penetrate into cerebellar tissue. Within the cerebellum, glutamine and glutamate, combining with NPs, inhibit intracellular antioxidant defenses. This suppression is featured by a decreased expression of GPX4 protein and reduced levels of GSH, leading to continuous accumulation of the lipid peroxidation product, MDA. Furthermore, this synergistic effect promotes iron release and exacerbates lipid peroxidation by upregulating NCOA4 and ALOX1 expression, thereby intensifying ferroptosis and ultimately leading to cellular structural damage and neuronal dysfunction in the cerebellum [90].

Recent studies have also pointed out that the core circadian clock transcription factor Bmal1 may play a crucial role in NPs exposure induced ferroptosis. Although the regulatory function of Bmal1 in neurons remains incompletely understood, some studies in lung tissue have confirmed that NPs exposure disrupts intracellular redox homeostasis through the Bmal1/Nrf2/HO-1 antioxidant pathway, triggering excessive ROS accumulation, lipid peroxidation, and iron metabolism dysregulation. Concurrent downregulation of GPX4 and upregulation of ACSL4 synergistically drive ferroptosis [91]. This mechanism may contribute to neurotoxicity through the lung–brain axis by reducing the expression of Bmal1. Specifically, decreased Bmal1 expression impaired the rhythmicity regulation of the Wnt signaling pathway, which plays a crucial role in neuronal regeneration and repair. Damage to Wnt signaling caused by Bmal1 suppression may affect neuronal regenerative and repair, thereby exacerbating neurotoxicity [92,93].

Furthermore, as a core regulator of circadian rhythms, the reduced expression of Bmal1 leads to circadian disruption. Many studies have shown that disruption of the circadian rhythm is closely associated with various neurodegenerative diseases, including Parkinson’s disease and Alzheimer’s disease [94]. Therefore, Bmal1 deficiency in the lung may indirectly aggravate neurotoxicity in the brain by disrupting systemic circadian homeostasis.

### 5.4. Autophagy

Autophagy is a highly conserved intracellular degradation and recycling process that eliminates damaged organelles, misfolded proteins, and invading pathogens via the lysosomal pathway to maintain cellular homeostasis and adapt to environmental stress. This process plays a crucial role in cell survival, development, and disease regulation, particularly in protecting neurons within the central nervous system [95,96]. As is known, autophagy regulates the cellular fate of both survival and death. Autophagy sustains the stability and integrity of the intracellular environment through eliminating intracellular damaged organelles and misfolded proteins. However, under certain pathological conditions or prolonged stress, autophagy-related cell death may be initiated [97]. Many previous studies suggest that autophagic dysregulation induced by environmental stressors contributes to the onset and progression of neurodegenerative diseases [98].

**Figure 4:**
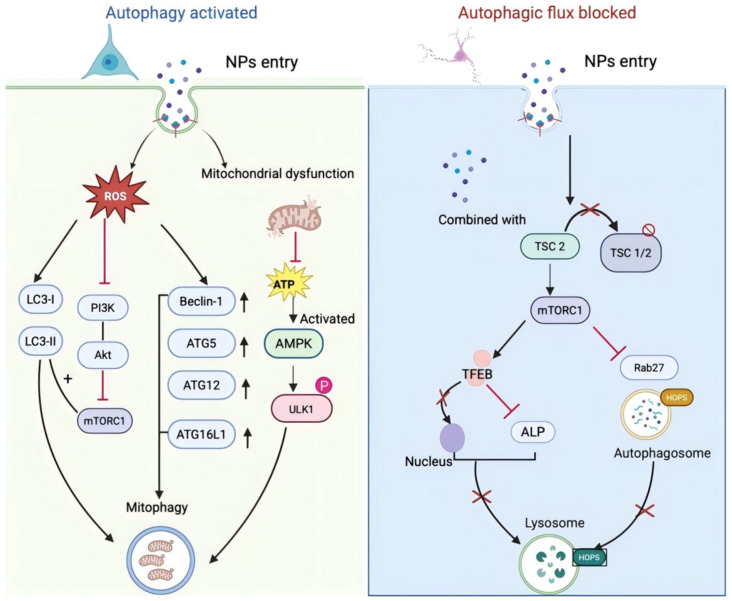
Effects of NPs exposure on autophagy and autophagic flux. (**Left**): NPs entry leads to the accumulation of reactive oxygen species (ROS), which in turn upregulates autophagy-related proteins, including LC3-I/II, Beclin-1, ATG5, ATG12, and ATG16L1, thereby activating autophagy; (**Right**): PS-NPs bind to TSC2, disrupting the TSC1/2 complex and consequently activating mTORC1. This suppresses the nuclear translocation of TFEB, leading to impaired autophagy–lysosome pathway (ALP) activity and blocked autophagic flux. Image created with BioRender.com. Wan, F. (https://BioRender.com/by15qsx) is licensed under CC BY 4.0.

Studies reveal that exposure to NPs regulates autophagy in a cell type-dependent manner. In human neuroblastoma SH-SY5Y cells, exposure to NPs at concentrations exceeding 100 μg/mL significantly increases intracellular ROS levels, which can initiate autophagy as well as a cascade of intracellular signaling events. Following NPs exposure, LC3-II, a canonical marker for autophagy activation, is markedly elevated in SH-SY5Y cells. In addition to LC3-II, the expression of autophagy-related proteins, such as Beclin-1 and the Atg5/12/16L1 complex, is upregulated as well, further confirming that exposure to NPs promotes autophagosome formation [99] (Figure 4 Left).

NPs exposure in MN9D cells disrupts autophagy through a distinct molecular mechanism. One study revealed that NPs bind to TSC2 protein, leading to dissociation of the TSC1-TSC2 complex that is crucial for maintaining mTOR activity homeostasis. As TSC2 negatively regulates the mTOR pathway, TSC2 dissociation from the TSC1-TSC2 complex induced by NPs causes aberrant mTOR hyperactivation. Hyperactivated mTOR inhibits the nuclear translocation of TFEB—a pivotal regulator of the autophagy–lysosome pathway (ALP). Impaired TFEB nuclear translocation suppresses ALP activation, prevents efficient autophagosome–lysosome fusion, and ultimately blocks autophagic flux. Additionally, NPs can impair autophagy via the lysosomal membrane permeabilization (LMP)-mediated autolysosome dysfunction. Accumulation of 30 nm NPs in the lysosomes of NSCs induces LMP by disrupting the F-actin cytoskeleton, leading to cathepsin B leakage into the cytosol and further inhibition of autophagic flux [100]. It is noteworthy that NP-mediated lysosomal dysfunction can also lead to the accumulation of misfolded proteins (p-PERK, XBP1s, CHOP), triggering endoplasmic reticulum stress. The excessive activation of misfolded proteins can disrupt proteostasis, thereby causing neurotoxic damage [101] (Figure 4 Right).

Although regulations of NPs-induced autophagy exhibit diversity in different cell lines, it is evident that NPs exposure disrupts the dynamic balance of cellular autophagy. Importantly, NPs-induced autophagic regulation does not strictly obey a simple “activation or inhibition” pattern but instead occurs sequentially. Initially, NPs exposure mediates the AMPK/ULK1 pathway to activate a protective autophagy reflection. However, following long-term exposure to NPs, lysosomal dysfunction (e.g., LMP or TFEB inhibition) blocks autophagic flux. Ultimately, this autophagic dysregulation, in concert with inflammasome activation, contributes to neuronal cell death.

Mitophagy plays an indispensable role in ensuring normal cellular physiology and intracellular environmental stability and is critically involved in neurodegenerative diseases and neuroinflammation [102,103]. In Alzheimer’s disease (AD), mitochondrial dysfunction is a hallmark pathological feature, as Aβ and hyperphosphorylated Tau interact with mitochondrial proteins to impair mitophagy, whereas enhancing mitophagy can suppress Aβ/Tau aggregation and reverse cognitive deficits in AD models [104]. Parkinson’s disease (PD) is characterized by dopaminergic neuron loss in the substantia nigra, a process closely associated with mitophagy impairment. Mutations in the PINK1/Parkin severely compromise the elimination of damaged mitochondria, thus leading to their accumulation and triggering a cascade of pathological events that ultimately initiate PD progress [105]. Studies demonstrate that NPs accumulating in cells directly target mitochondrial complex I, leading to energy metabolism disturbances, which subsequently activate the AMPK/ULK1-PINK1/Parkin signaling pathway, triggering excessive mitophagy, ultimately resulting in dopaminergic neuron death [102]. Taken together, one can assume from this that NPs may disturb intracellular mitochondrial homeostasis during the progress of neurodegenerative diseases [105].

### 5.5. Effects of Exposure to NPs on the Gut–Brain Axis

The digestive tract is well known to serve as the primary route for NPs exposure; it has been reported that nanoparticles are detectable in human feces [106]. Many studies indicate that gut microbiota dysbiosis induced by NPs may modulate the host immune system via gut metabolic pathways, thereby triggering neuroinflammation through the gut–brain axis.

Research has reported that maternal exposure to NPs disrupts intestinal microbial homeostasis in offspring mice, characterized by a reduction in beneficial anti-inflammatory bacteria, including *Lachnoclostridium*, *Lachnospiraceae*_NK4A136 and *Odoribacter* populations, whereas there is an increased abundance of pro-inflammatory taxa such as *Alistipes* and the *family* XIII AD3011 populations. It is noteworthy that the small size characteristics of nanoplastics can directly damage the cell membranes of microbiota and alter their morphology and function, leading to a reduction in the structural diversity of microbial communities [107]. Additionally, pathogenic *Verrucomicrobiota* populations expand while beneficial *Patescibacteria* are markedly diminished. This intestinal dysbacteriosis contributes to neuroinflammation by disrupting immune regulation and gut metabolic function. For instance, diminishment in *Patescibacteria* impairs mitochondrial function and host metabolism, thereby initiating chronic neuroinflammation through fatty acid-mediated inflammatory pathways [108].

Short-chain fatty acids (SCFAs) can enter the brain through the bloodstream, where SCFAs are an energy source for neural cells and suppress microglia activation, thereby performing anti-inflammatory function [109]. SCFAs deficiency results in insufficient energy supply in the brain and loss of microglia suppression, making the brain more susceptible to neuroinflammation [110]. Exposure to NPs may reduce the abundance of *Bifidobacterium* and *Lactobacillus*, subsequently causing a decrease in SCFAs in animal models. In addition to producing SCFAs via carbohydrates fermentation, *Bifidobacterium* genus can participate in the tryptophan metabolism, promoting the synthesis and production of serotonin through specific enzymatic reactions [111]. Metabolites produced during tryptophan metabolism, as well as SCFAs produced by *Bifidobacterium* genus, exhibit significant neuroprotective effects, effectively mitigating neurotoxicity [112]. Therefore, exposure to NPs may increase the susceptibility of neuronal cells to neuroinflammation by disturbing microbial homeostasis to reduce the production of SCFAs, which are important neuroprotective gut metabolites.

NPs exposure increases the abundance of *Desulfovibrio* in the intestine. Studies have shown that the quantity of *Desulfovibrio* in human fecal samples positively correlates with Parkinson’s disease severity. *Desulfovibrio* utilizes sulfate to produce hydrogen sulfide (H_2_S), which promotes the aggregation of α-synuclein (α-SYN) [112]. Moreover, H_2_S can enter the central nervous system via the circulatory system, then inhibit mitochondria respiration in neuronal cells, and disturb neuronal energy metabolism. When combined with NPs exposure, these two effects act synergistically to further exacerbate neurotoxicity and contribute to cognitive dysfunction. In addition to H_2_S, *Desulfovibrio* can produce bacterium magnetite particles that may translocate from the gut into the bloodstream, cross the BBB, and accelerate α-SYN aggregation in the brain, thereby contributing to neurodegenerative processes [113]. NPs exposure also disrupts the metabolic balance of the Bacteroides genus, leading to its excessive proliferation. Overgrown Bacteroides produce trimethylamine (TMA) during the metabolic process, which is subsequently oxidized and converted to trimethylamine N-oxide (TMAO). The accumulation of TMAO in organs, such as the liver, induces excessive ROS production, which disrupts neuronal redox balance and triggers ferroptosis, thereby significantly aggravating the neurotoxic damage induced by NPs exposure [114].

According to current understandings, modulation of the gut microbiota may be a potential strategy to alleviate the neurotoxicity induced by NPs. For instance, *Lactobacilli* strains DT22 and DT66 have been shown to significantly increase the *Firmicutes*/*Bacteroidetes* (F/B) ratio in mice exposed to NPs. The F/B ratio serves as a key indicator of gut microbiota balance; its appropriate elevation promotes the development, differentiation and activation of intestinal immune cells, enhances intestinal immune responses, and may further modulate systemic immunity via the gut–brain axis [115].

In addition to disturbing microbiota homeostasis, NPs exposure can also induce neuroinflammation by impairing intestinal barrier integrity. Histological analyses in studies show that after mice are exposed to NPs exposure causes intestinal epithelium damage, featuring partial loss of intestinal villi, disorganization of the lamina propria, reduction of mucus coverage, decreased mucin, and tight junction proteins expression. Impaired intestinal barrier integrity facilitates bacterial endotoxins, such as lipopolysaccharide (LPS), to leak into the bloodstream, thereby activating neuroinflammatory response as well as a systemic inflammatory response. Studies have revealed that Nrf2 signaling may be involved in a neuroinflammatory process induced by NPs exposure. Compared with wild-type mice, intestinal epithelial-specific Nrf2 knockout mice (Nrf2fl/fl-VilCre+) exhibit an increased expression of Th17-associated markers, in particular, a significant increase in IL-17C mRNA levels. Excessive intestinal IL-17C may affect the systemic circulation and potentially contribute to neuroinflammatory processes, suggesting a possible gut–brain inflammatory signaling pathway.[54]

In summary, exposure to NPs disrupts gut microbiota composition, promotes the proliferation of pathogenic bacteria, impairs intestinal barrier integrity, and activates pro-inflammatory immune pathways. Through these interrelated mechanisms, NPs exposure initiates a neuroinflammatory response via the gut–brain axis, representing a critical pathway underlying their neurotoxic effects.

## 6. NPs Exposure and Neurodegenerative Diseases

In recent years, the incidence of major neurodegenerative diseases, represented by Alzheimer’s disease (AD) and Parkinson’s disease (PD), has shown a significant upward trend, becoming a major public health challenge for aging societies worldwide [116]. Epidemiological studies reveal that a considerable proportion of sporadic cases cannot be fully attributed to genetic susceptibility but are closely associated with environmental exposure. Among these, NPs, as a prevalent yet long-overlooked class of emerging environmental pollutants, have attracted extensive attention in the field of neurotoxicology due to their unique physicochemical properties and potential neurotoxic effects.

### 6.1. Mechanistic Differences Between Nanoplastics and Inorganic Particles in Inducing Neurodegenerative Diseases

Compared to traditional inorganic nanoparticles, such as TiO_2_ and SiO_2_, NPs exhibit distinctly different biological interaction characteristics in biological fluids. The highly hydrophobic and chemically inert surface of NPs enables rapid adsorption of plasma proteins to form a protein corona, enabling them to evade immune recognition [117]. Specifically, NPs preferentially enrich anti-opsonins, such as apolipoprotein E (ApoE), clusterin, and vitronectin, and evade immune surveillance through the following multiple mechanisms: (1) inhibiting the complement cascade, blocking the formation of C3 convertase and assembly of the membrane attack complex (MAC); (2) competitively occupying phagocyte surface receptors, interfering with Fc receptor- and complement receptor-mediated recognition; and (3) mimicking the “self” signal of high-density lipoprotein (HDL) to suppress the clearance function of the mononuclear phagocyte system [118,119,120]. The NPs that escaped successfully cross the blood–brain barrier (BBB) via transcytosis, especially LRP1 receptor-mediated vesicular transport, and accumulate in the central nervous system to induce neurotoxicity. Conversely, the protein corona built on the surface of inorganic nanoparticles is generally enriched with opsonins, such as immunoglobulins, and complement proteins, which instead promote recognition and clearance by the mononuclear phagocyte system, which limits their bioaccumulation in the brain [121].

Furthermore, the environmental aging process of NPs amplifies their neurotoxic risks. When NPs undergo photo-oxidation or thermal aging in the environment, their surfaces undergo chemical modifications, generating oxygen-containing functional groups, such as carbonyl (C=O) and hydroxyl (-OH), and producing environmentally persistent free radicals (EPFRs). These EPFRs can persist in organisms for weeks to months, continuously inducing oxidative stress through Fenton reactions and the Haber–Weiss cycle, selectively disrupting the homeostasis of neurotransmitter systems, including dopamine, glutamate, and GABA [122]. In stark contrast, inorganic nanoparticles (such as TiO_2_) require specific UV wavelengths to activate their photocatalytic properties [123]. Within biological systems, these particles can be gradually degraded through lysosomal enzymatic breakdown or chemical transformation, resulting in toxicity that diminishes over time rather than accumulating.

In summary, there are fundamental differences in the mechanisms of interaction between NPs and inorganic nanoparticles with organisms: the immune evasion properties of the protein corona, the ability to penetrate the BBB, and the sustained oxidative stress effects after environmental aging collectively constitute the basis for the unique neurotoxic characteristics of NPs. These differences may be key to explaining why NPs can induce neurodegenerative disorders at environmentally relevant concentrations, whereas inorganic particles require higher exposure doses.

### 6.2. Alzheimer’s Disease

Alzheimer’s disease (AD), the most prevalent and burdensome neurodegenerative disorder worldwide, is comprised of over 90% sporadic cases. Its pathological progression results from long-term interactions between genetic susceptibility (e.g., APOE ε4 allele) and various environmental factors [124]. In recent years, multiple independent studies—spanning population-based epidemiological associations, rodent pathological models, and human postmortem neuropathological evidence—have consistently demonstrated a strong link between NPs exposure and characteristic AD pathological changes. These findings provide multidimensional scientific support for NPs as a potential environmental risk factor for AD.

At the epidemiological level, dietary exposure studies provide environmental clues. A positive correlation exists between high consumption of processed foods (the primary source of oral exposure to NPs) and increased risk of AD onset [125]. Although this association is influenced by multiple confounding factors, such as dietary patterns, metabolic syndrome, and socioeconomic status, it still offers preliminary population-level evidence supporting the causal hypothesis of “nanoplastic environmental exposure-neurodegenerative diseases”.

From the perspective of molecular pathological mechanisms, NPs accelerate the two core pathological events of AD—amyloid deposition and neurofibrillary tangle formation—through multi-target interference. Specifically, the accumulation of NPs in the brain can significantly promote the nucleation process of amyloid-β (Aβ) and its key subtypes, Aβ40 and Aβ42 [126]. NPs facilitate the creation of Aβ oligomers, which are toxic, through adsorption on hydrophobic surfaces, which increases the deposition of amyloid plaques in the brain parenchyma. Different types of polymer NPs exhibit different neurotoxicities. Polystyrene nanoplastics promote the elongation and crosslinking of Aβ fibrils, polyethylene/polypropylene NP may cause neural damage by enriching toxic soluble oligomer through inhibiting fibril growth and enhancing soluble toxic oligomer accumulation [127].

In addition, NP exposure can enhance the hyperphosphorylation and aggregation of tau protein by activating tau kinases such as glycogen synthase kinase-3β (GSK-3β) and cyclin-dependent kinase 5 (CDK5). Hyperphosphorylated tau protein loses its ability to stabilize microtubules and spontaneously assembles into neurofibrillary tangles (NFTs), leading to impaired axonal transport, synaptic loss, and neuronal death [11]. This dual pathological hit of “amyloid cascade-neurofibrillary tangles” collectively constitutes the molecular basis of NPs-induced neurodegenerative changes in AD.

### 6.3. Parkinson’s Disease

Parkinson’s disease (PD), the second most prevalent neurodegenerative disorder after Alzheimer’s disease, is characterized by the selective degeneration and death of dopaminergic neurons in the substantia nigra of the midbrain and the depletion of dopamine neurotransmitters in the striatum [128]. Current studies have revealed that exposure to nanoplastics (NPs) can induce the onset and progression of PD through multi-level molecular mechanisms. At the cytotoxic level, NPs primarily trigger mitochondrial dysfunction, leading to the collapse of energy metabolism in dopaminergic neurons and oxidative stress-mediated cell death. Simultaneously, NPs activate the NF-κB signaling pathway in microglia, initiating a neuroinflammatory cascade that releases pro-inflammatory factors, such as TNF-α and IL-1β, further exacerbating neuronal damage [129]. At the pathological protein level, NPs can promote the pathological aggregation of α-synuclein through hydrophobic interactions and inhibit lysosomal degradation function, leading to the formation of Lewy body-like inclusions [130]. Additionally, NPs disrupt the integrity of the intestinal mucosal barrier and disturb gut microbiota homeostasis (e.g., reducing the abundance of Prevotellaceae and *Faecalibacterium*), thereby facilitating the trans-systemic spread of pathological proteins via the gut–brain axis and indirectly regulating the onset of PD.

In terms of trans-barrier transport and bioaccumulation, animal studies have demonstrated that fluorescent signals can be detected in brain parenchyma (including the substantia nigra pars compacta) as early as 1.5 h after oral exposure to PS-NPs, confirming their rapid penetration across the BBB [100]. This finding is further supported by clinic–pathological evidence: recent detections of nanoplastic particles in brain tumor patients and postmortem brain tissues indicate that the human BBB cannot effectively block nanoscale plastic particles, enabling their long-term accumulation in the central nervous system and the induction of neurotoxicity [131].

At the molecular regulatory level, studies have confirmed that NPs regulate the autophagy function of dopaminergic neurons through the TSC2-mTOR-TFEB signaling axis. Specifically, NPs exposure significantly downregulates the expression of tuberous sclerosis complex 2 (TSC2), thereby relieving its inhibitory effect on mTORC1. This leads to excessive mTOR activation and phosphorylation of transcription factor EB (TFEB), preventing its nuclear translocation and ultimately suppressing lysosome biogenesis and autophagic flux. Notably, this finding aligns with previous autopsy studies—significant reductions in TSC2 protein levels have been observed in both PD patient brain tissues and classic PD animal models, suggesting that dysregulation of the TSC2-mTOR-TFEB pathway may serve as a key molecular hub connecting environmental NP exposure to PD pathogenesis [100]. In summary, NPs promote the onset and progression of PD against a backdrop of genetic susceptibility through multiple mechanisms, including “protein aggregation catalysis, energy metabolism impairment, neuroinflammatory amplification, and autophagy clearance suppression”.

### 6.4. Amyotrophic Lateral Sclerosis

Amyotrophic lateral sclerosis (ALS) is a fatal neurodegenerative disease that causes progressive degeneration and the death of motor neurons in the anterior horn of the spinal cord and the cerebral cortex. The 43 kDa TAR DNA-binding protein (TDP-43) demonstrates abnormal metabolism at the molecular pathological level. TDP-43 is a marker of ALS. A total of 97% of ALS patients exhibit cytoplasmic aggregation and nuclear depletion of TDP-43 in the brain and spinal cord. In disease conditions, TDP-43 is often subject to several post-translational modifications, such as hyperphosphorylation and ubiquitination, which hinder its nucleocytoplasmic transport functions and result in the assembly of cytotoxic membraneless organelle-like aggregates in the cytoplasm. According to research findings, there has been a growing interest in exploring the link between sporadic ALS and environmental exposures. These include the possible neurotoxicity of nanoparticles [132,133].

The current evidence indicates that NPs in motor neurons may modulate the disease, although it has not been elucidated that NPs directly cause ALS. However, NPs certainly have the capacity to destabilize RNA-binding protein homeostasis, leading to disease-modulating output. NPs are able to pass the blood–brain barrier and enter the central nervous system through the circulation or retrograde axonal transport of neurons. NPs will consequently accumulate in the cell body of motor neurons, resulting in an oxidative stress microenvironment. The oxidative stress caused by NPs at the molecular mechanism triggers the TDP-43 pathologic event as the key event. In particular, the activation of reactive oxygen species interferes with the oxidative modification and subsequent inactivation of the cysteine residues of HSP70. As an essential molecular chaperone for TDP-43 nuclear import, oxidatively inactivated HSP70 is unable to recognize and bind to the TDP-43 nuclear localization signal (NLS), leading to cytoplasmic retention in TDP-43. Most importantly, mislocalized TDP-43 is quelled by NPs due to the hydro-phobicity of their surfaces, forming NPs-TDP-43 complexes. The complex functions as a ‘pathological seed’, which drives the TDP-43 protein to undergo abnormal condensation through liquid–liquid phase separation, gelation/solidification, eventually forming irreversible amyloid aggregates [25]. These solid aggregates are thought to lose their normal regulatory functions towards RNA. They may recruit other RNA-binding proteins via a ‘sequestration’ mechanism, disrupting stress granule assembly [134]. Ultimately, this process triggers pathways of apoptotic or necrotic cell death, resulting in the deterioration of motor neurons, progressively. In a nutshell, NPs recapitulate the core pathological features of ALS at cellular and molecular levels through the cascade mechanism of oxidative stress-HSP70 inactivation-TDP-43 nucleocytoplasmic transport impairment and pathological phase separation solidification, with environmental nanoplastics exposure being a possible risk factor for sporadic ALS.

### 6.5. Huntington’s Disease

Huntington’s disease (HD) is an autosomal dominant polyglutamine (polyQ) disorder caused by the abnormal expansion of CAG trinucleotide repeats in exon 1 of the HTT gene. The neuropathological hallmark manifests as elongation of the N-terminal polyQ chain in mutant huntingtin (mHTT), leading to protein misfolding and formation of insoluble aggregates within neuronal nuclei and cytoplasm [135]. This subsequently triggers selective degeneration and death of medium spiny neurons (MSNs) in the striatum. At the molecular level, the pathological cascade of HD involves multisystem dysfunction, including: decreased activity of mitochondrial electron transport chain complexes and reduced oxidative phosphorylation efficiency, collapse of the proteostasis network in the ubiquitin–proteasome system (UPS) and autophagy–lysosome pathway (ALP); as well as oxidative stress damage mediated by impaired copper ion homeostasis in the brain [136,137].

Although current epidemiological and experimental studies lack direct causal evidence linking nanoplastics (NPs) to the induction of Huntington’s disease (HD), the potential for NPs to exacerbate HD progression as an environmental risk factor warrants significant attention, given the substantial overlap between their well-documented neurotoxic mechanisms and HD pathological pathways. Specifically, NPs can penetrate the blood–brain barrier and accumulate in deep brain regions, such as the striatum, potentially synergizing with mHTT toxicity to exacerbate damage. Furthermore, NPs may act as carriers for metal ions, particularly copper, enriching its levels and catalyzing the covalent crosslinking and aggregation of mHTT through redox cycling, while also inducing copper-dependent cell death (cuproptosis) [138]. Given the significant individual variations in the age of onset and progression rate of sporadic HD, coupled with the interaction between environmental exposure and genetic background, the chronic damage of NPs to mitochondrial function and protein clearance systems may serve as a “second hit” to accelerate disease onset or exacerbate motor and cognitive phenotypes in genetically susceptible individuals. Therefore, systematic in vitro and in vivo toxicological assessments targeting the specific toxic mechanisms of NPs in neurodegenerative diseases and their interaction with mHTT aggregation hold significant public health implications and preclinical research value.

In summary, although neurodegenerative diseases, such as Alzheimer’s disease, Parkinson’s disease, amyotrophic lateral sclerosis, and Huntington’s disease, exhibit significant differences in characteristic pathological proteins (e.g., Aβ, α-synuclein, TDP-43, mHTT) and selectively affected neuron types, current evidence suggests that exposure to nanoplastics (NPs) appears to be involved in the pathological processes of multiple neurodegenerative disorders through convergent mechanisms. The core of these shared mechanisms lies in oxidative stress-driven proteostasis imbalance: NPs induce reactive oxygen species (ROS) burst, inhibit proteasome and autophagy–lysosome pathway functions, and promote pathological protein misfolding, oligomerization, and fibril formation. Simultaneously, NPs act as “seed templates” to accelerate protein aggregation kinetics through hydrophobic interactions, while disrupting mitochondrial energy metabolism and lysosomal acidification, forming a vicious cycle of “oxidative stress-protein aggregation-clearance impairment”. Furthermore, NPs activate neuroinflammatory responses in microglia and astrocytes, amplifying neuronal damage. This inflammation–degeneration cascade exhibits high conservation across different diseases.

However, current research on the causal relationship between NPs exposure and neurodegenerative diseases still exhibits significant limitations, hindering the translation from experimental observations to population risk assessment. The primary bottleneck lies in the inadequate extrapolation of study models: existing evidence predominantly relies on acute or sub-chronic animal experiments (e.g., mice, zebrafish, *C. elegans*) and in vitro cell culture systems. These models struggle to simulate the chronic effects of long-term, low-dose, multi-route (inhalation, ingestion, dermal contact) exposure in humans. Moreover, the administered doses (typically at mg/kg levels) often far exceed actual environmental exposure levels (ng-μg/kg range), resulting in uncertainties in dose–response relationships.

Human epidemiological studies are constrained by methodological challenges in exposure assessment (lack of standardized biomarkers) and interference from long disease latency periods, making it difficult to establish reliable temporal–causal inferences. It is noteworthy that, although the dual-injection pyrolysis–gas chromatography/mass spectrometry (Py-GC/MS) technique has enabled semi-quantitative analysis of thermal degradation products from plastic polymers in blood, thereby allowing for the estimation of mass concentrations for various polymers, this method has inherent limitations: it can only provide total mass information of polymers, failing to capture specific particle counts, size distribution, or morphological characteristics. This makes it difficult to comprehensively characterize the spatial distribution of plastic particles in blood, their biotransformation status, and interaction patterns with biomolecules, ultimately limiting the transition from “mass load” to “particle dose-effect” risk assessment. Currently, the quantitative detection of NPs in humans still faces multi-dimensional technical challenges, involving a series of core issues such as sample complexity, particle heterogeneity, detection sensitivity, and a lack of standardization. Future breakthrough directions can be advanced synergistically through two levels: direct detection and indirect biomarkers. At the direct detection level, it is necessary to develop asymmetric flow field-flow fractionation (AF4) coupled with mass spectrometry for the simultaneous analysis of particle size and chemical composition, as well as single-particle characterization methods, such as surface-enhanced Raman scattering (SERS) and nano-secondary ion mass spectrometry (NanoSIMS), to achieve high-throughput and high-resolution detection of trace NPs. At the indirect biomarker level, specific biological responses induced by NP exposure can be identified such as oxidative stress markers (8-hydroxy-2′-deoxyguanosine, 8-OHdG), systemic inflammatory factors (IL-6, TNF-α), and post-translational protein modifications (protein carbonylation, nitration). Additionally, immunoaffinity-mass spectrometry methods targeting NP-pathological protein complexes (e.g., NPs-α-synuclein, NPs-Aβ, NPs-TDP-43) should be developed. These complexes not only serve as direct evidence that NPs can penetrate the blood–brain barrier and participate in neurodegenerative pathologies but may also function as biomarkers for early neurotoxic effects. This provides crucial technical support for establishing the risk assessment chain of “environmental exposure-biomarker-clinical outcome”.

## 7. Discussion

Numerous studies have shown that the neurotoxicity of nanoplastics is not due to a single mechanism of action, but rather the combined effects of several interlinked and entwined complex pathways. There is a lot of crosstalk and synergy, meaning that none of these mechanisms act in isolation; they all compound the damage that neural tissues sustain. Oxidative stress, as an example, directs damage to neuronal lipids, proteins, and DNA; it is not restricted to this, as it also activates the neuroinflammatory pathways triggering excessive microglial activation and releases large quantities of pro-inflammatory factors such as TNF-α, IL-1β and IL-6. The oxidative stress responses are further amplified by these inflammatory factors. The joint effect of neuroinflammation and oxidative stress may impair autophagosome–lysosome fusion. When cells are stimulated by NPs, there may be interference in the formation of autophagosomes or their fusion with lysosomes, and damaged organelles and misfolded proteins are ineffective. This can result in disturbances in the intracellular environment and even neuronal apoptosis or ferroptosis. Ferroptosis is a kind of cell death driven by lipid peroxidation. It is iron-dependent and associated with oxidative stress. Nanoparticles have the potential to disrupt the homeostasis of intracellular iron ions, resulting in the release of free iron and inducing lipid peroxidation through the Fenton reaction. This process eventually causes neuronal death.

In addition, the gut–brain axis, as a regulatory pathway that has garnered significant attention in recent years, may first be compromised by NPs exposure, leading to the disruption of intestinal barrier integrity, imbalance in gut microbiota structure, and abnormal metabolic products. These alterations can affect the central nervous system through various pathways, such as blood circulation, neural signaling, or immune regulation, thereby amplifying neurotoxic effects. The synergistic action of these multiple mechanisms renders the neurotoxicity of nanoplastics both complex and diverse, posing substantial challenges for comprehensively understanding their potential hazards and developing effective protective strategies. Therefore, future research needs to systematically and comprehensively investigate the interaction networks among these mechanisms, as well as their dynamic changes and dominant roles under different exposure conditions (e.g., types of NPs, concentration, duration, and routes of exposure). This will help to more thoroughly reveal the essence of nanoplastics neurotoxicity and provide important theoretical foundations for developing targeted interventions.

Based on existing studies, the potential hazards of NPs to the nervous system have become increasingly evident. Their toxic effects not only involve direct cellular damage, such as oxidative stress and ferroptosis, but also exert indirect influences through complex regulatory networks like the gut–brain axis. This multi-pathway, multi-target toxicity profile makes assessing the neuro-safety of NPs particularly challenging. Currently, most research remains focused on exploring single mechanisms or observing toxic effects under specific exposure conditions. There is still insufficient in-depth understanding of how different mechanisms crosstalk and synergistically amplify toxicity, as well as the dynamic evolution patterns and key driving factors of these mechanisms under real-world scenarios of low-dose, long-term exposure. For example, whether NP-induced oxidative stress further exacerbates iron ion dysregulation, or how specific metabolites produced by gut microbiota imbalance precisely regulate inflammatory responses in the central nervous system remain to be elucidated. Furthermore, different types of NPs (such as variations in size, surface modification, and chemical composition) may exhibit significant differences in their manifestations and in the intensity of neurotoxicity. However, existing studies often employ a limited selection of NPs, making it difficult to comprehensively reflect the complexity of NP exposure in real-world environments. Additionally, more in vitro and in vivo experimental evidence is needed to elucidate the specific mechanisms by which NPs cross the blood–brain barrier, their accumulation patterns in the brain, and their potential associations with the onset and progression of neurodegenerative diseases. Therefore, future research should focus on developing experimental models that more closely mimic real-world exposure scenarios. By integrating multi-omics technologies (such as transcriptomics, metabolomics, metagenomics, etc.), we can systematically analyze the molecular networks and signaling pathways underlying NPs’ neurotoxicity, identifying key regulatory nodes and biomarkers. This approach will not only provide a more scientific theoretical basis for the risk assessment of nanoplastics’ neurotoxicity but also open new avenues for developing effective prevention and intervention strategies to safeguard human neurological health.

## 8. Conclusions

In conclusion, accumulating evidence indicates that NPs exposure exerts significant neurotoxic effects, which are manifested by behavioral impairments and neuropathological changes. These adverse effects are mediated through multiple, interrelated mechanisms, including oxidative stress, neuroinflammation, ferroptosis, autophagy dysfunction and disruption of the gut–brain axis. Exposure to NPs has also been implicated in the initiation and progression of neurodegenerative diseases, such as Alzheimer’s disease and Parkinson’s disease, largely by the promotion of aberrant protein aggregation and progressive neuronal damage. In spite of emerging advances in the effects of NPs exposure on the nerve system, the current understanding is predominantly derived from experimental models; the extent to which these findings can be extrapolated to human health remains uncertain. Moreover, the long-term consequences of chronic, low-dose exposure to NPs under environmentally relevant conditions still need to be further clarified. Therefore, future studies should focus on elucidating the interactive effects between NPs and other environmental pollutants, as well as unraveling the precise molecular and cellular pathways underlying NPs-induced neurotoxicity. Such efforts are essential to establish the molecular mechanisms that form the theoretical basis for risk assessment and preventive strategies aimed at mitigating the neurological hazards of nanoplastics exposure.

## Data Availability

No new data were created or analyzed in this study. Data sharing is not applicable to this article.
